# The Use of a Polyphenoloxidase Biosensor Obtained from the Fruit of Jurubeba (*Solanum paniculatum* L.) in the Determination of Paracetamol and Other Phenolic Drugs

**DOI:** 10.3390/bios8020036

**Published:** 2018-04-02

**Authors:** Rafael Souza Antunes, Luane Ferreira Garcia, Vernon Sydwill Somerset, Eric de Souza Gil, Flavio Marques Lopes

**Affiliations:** 1 Faculdade de Farmácia, Universidade Federal do Goiás (UFG), rua 221 esquina com a 5ª avenida s/n, Setor Universitário, Goiânia-GO 74605-170, Brazil; rafael.antunes@panpharma.com.br (R.S.A.); luane.fg@hotmail.com (L.F.G.); 2 Department of Chemistry, Faculty of Applied Sciences, Cape Peninsula University of Technology, Bellville 7535, South Africa; vsomerset@gmail.com

**Keywords:** plant enzymes, vegetable polyphenoloxidases, amperometric biosensors, pharmaceutical analysis

## Abstract

The vegetable kingdom is a wide source of a diverse variety of enzymes with broad biotechnological applications. Among the main classes of plant enzymes, the polyphenol oxidases, which convert phenolic compounds to the related quinones, have been successfully used for biosensor development. The oxidation products from such enzymes can be electrochemically reduced, and the sensing is easily achieved by amperometric transducers. In this work, the polyphenoloxidases were extracted from jurubeba (*Solanum paniculatum* L.) fruits, and the extract was used to construct a carbon paste-based biosensor for pharmaceutical analysis and applications. The assay optimization was performed using a 0.1 mM catechol probe, taking into account the amount of enzymatic extract (50 or 200 μL) and the optimum pH (3.0 to 9.0) as well as some electrochemical differential pulse voltammetric (DPV) parameters (e.g., pulse amplitude, pulse range, pulse width, scan rate). Under optimized conditions, the biosensor was evaluated for the quantitative determination of acetaminophen, acetylsalicylic acid, methyldopa, and ascorbic acid. The best performance was obtained for acetaminophen, which responded linearly in the range between 5 and 245 μM (R = 0.9994), presenting a limit of detection of 3 μM and suitable repeatability ranging between 1.52% and 1.74% relative standard deviation (RSD).

## 1. Introduction

In the last decades, the use of plant tissues and vegetable enzymes for the development of analytical tools for pharmaceutical, food, and clinical analysis has received noticeable attention. In fact, owing to the laborious work of isolation and conditioning, the use of purified or commercial enzymes has become too expensive. Thus, the use of crude extracts or plant tissues is attractive, and also offers conditions closer to the optimum ones found in a natural medium, hence favoring the biocatalytic applications [1,2].

Moreover, the great advances of biotechnology and electrochemical sciences have allowed the fabrication of selective and suitable devices at low cost [3,4,5,6]. 

It should also be noted that the socioeconomic background and practicality of the green use of our great biodiversity in order to provide enzymatic materials is noteworthy [7]. Among the main classes, the polyphenol oxidases (PPO), that is, tyrosinase, phenolase, catechol oxidase, catecholase, and creolases, are copper enzymes that are widely found in plant tissues. Such enzymes are able to promote the hydroxylation of monophenols to produce ortho- or para-diphenolic compounds as well as remove hydrogen phenolic hydroxyl groups to produce the keto derivatives [8,9]. 

The jurubeba fruit (*Solanum paniculatum* L.) is a shrub-like solanaceae native to the Brazilian Cerrado that bear fruits all year [10]. Owing to their main phytochemicals, that is, antioxidants, steroids, saponins, alkaloids, and glycosides, all of pharmacological relevance, this plant is at the top of the list of the main Brazilian herbal medicines [8].

Furthermore, this plant is also a good source of polyphenol oxidase; thus, its tissue or crude extracts can be immobilized in electrochemical materials in order to obtainbiosensors. The easy and most convenient procedure of immobilization is the occlusion of carbon paste [11]. Polyphenol oxidase-based biosensors have been applied to the pharmaceutical analysis of phenolic drugs, including acetaminophen and acetyl salicylic acid analysis [12,13,14,15].

The analgesics acetaminophen (paracetamol) and acetyl salicylic acid (aspirin) are two of the most-consumed drugs around the world [16,17]. Therefore, a myriad of spectrophotometric [18], chromatographic [17], electrophoretic [19], and voltammetric [5] analytical proposals involving them can be found in the literature. 

The great appeal for the development and validation of new methods is driven by the requirement of faster and cheaper procedures that, in the case of pharmaceuticals, must keep the analytical standards in accordance with the rigorous regulatory issues. Thus, the use of plant enzyme-based biosensors can enable improved analysis since it provides speed, selectivity, and low cost [3,5,6].

Therefore, the aim of this work was the development of a carbon paste-based biosensor, in which the recognizing agent was polyphenoloxidases (PPOs) from a crude extract of jurubeba (*S. paniculatum* L.) fruits. The optimized biosensor was evaluated by quantitative determinations of paracetamol, aspirin^®^, methyldopa, and ascorbic acid by means of differential pulse voltammetry (DPV) assays. The best biosensor system employing the jurubeba fruit was determined in this study, following validation and comparison to the official regulations and pharmacopeial methods available [20,21,22,23].

## 2. Materials and Methods

### 2.1. Reagents and Solutions 

All electrolyte solutions were prepared using analytical standard salts from Vetec Química Fina Ltda. (Rio de Janeiro, Brazil), which were diluted in purified water, obtained from a Millipore Milli-Q purification system with conductivity ≤0.1 μS cm^−1^, Millipore S/A (Molsheim, France). The paracetamol and methyldopa standard were purchased from Sigma-Aldrich (St. Louis, MO, USA). Salicylic acid and ascorbic acid were donated by the University Pharmacy of the Federal University of Goiás (UFG), Goiânia-GO, Brazil. All solution standards were prepared at a concentration of 100 μM from the dilution of stock solutions (1 mM).

### 2.2. Plant Material and Preparation of the Raw Vegetable Extract

The jurubeba fruits were collected in August 2016 from a single collection and plant, located on the Rio das Almas in the city of Rialma, GO, Brazil; geographic coordinates: 15°19′08.65″ S 49°35′19.38″ W. Around 100 fruits were collected. After collection, the fruits were washed, packed in polyethylene bags, and stored for 3 days at 4 °C until analysis.

For the preparation of the raw vegetable extract, the jurubeba fruits were frozen for 24 h before being treated. After this period of freezing, they were processed in the Yononas^®^ appliance (Briton, Brazil). It was possible to obtain a frozen paste called “ICEjur”: jurubeba fruit ice cream. Then, 20 g of the ICEjur was diluted in 100 mL of 0.05 M sodium phosphate buffer (pH 6.0), homogenized for 10 min under stirring on a magnetic stirrer, and filtered on TNT fabric filter (nonwoven fabric), thereby obtaining a 20% crude vegetable extract (JEE) (pH 6.0). An ambient temperature of 20 °C ± 2 °C was applied.

### 2.3. PPO Enzymatic Activity Determination

In order to evaluate the PPO enzymatic activity, a spectrophotometric approach was used. To do so, 100 μL JEE and 3 mL 0.07 M catechol solution (in 0.05 M phosphate buffer (PB) solution, pH 6.0) were mixed, then after 10 min the absorbance value at 420 nm was measured, using an UV–visible spectrophotometer (Q798U2VS, Quimis Aparelhos Científicos Ltda., São Paulo, Brazil) [24]. The blank was set as 0.07 M catechol solution in 0.05 M PB solution (pH 6.0) with 100 μL JEE. The PPO activity was expressed in U/mg protein.

Protein content determination was performed according to the Bradford method [25], using bovine albumin serum (BSA) as a standard solution. In this case, 100 μL JEE and 5 mL Bradford reagent were mixed, then after 10 min the absorbance was measured at 595 nm. All experiments were performed in triplicate at room (20 ± 2 °C) temperature.

### 2.4. Assay Development: Biosensor, Effect of pH, and Electrochemical Parameters

The carbon paste was prepared using graphite powder and mineral oil, both from Sigma-Aldrich (St. Louis, MO, USA). The construction of the biosensors was carried out after enzymatic immobilization in JEE carbon paste by the physical adsorption technique (Table 1). The enzymatic extract was added directly to the graphite powder, which was homogenized and dried at room temperature (20 ± 2 °C). Subsequently, the mineral oil was added and the slurries were thoroughly mixed.

**Scheme 1:**
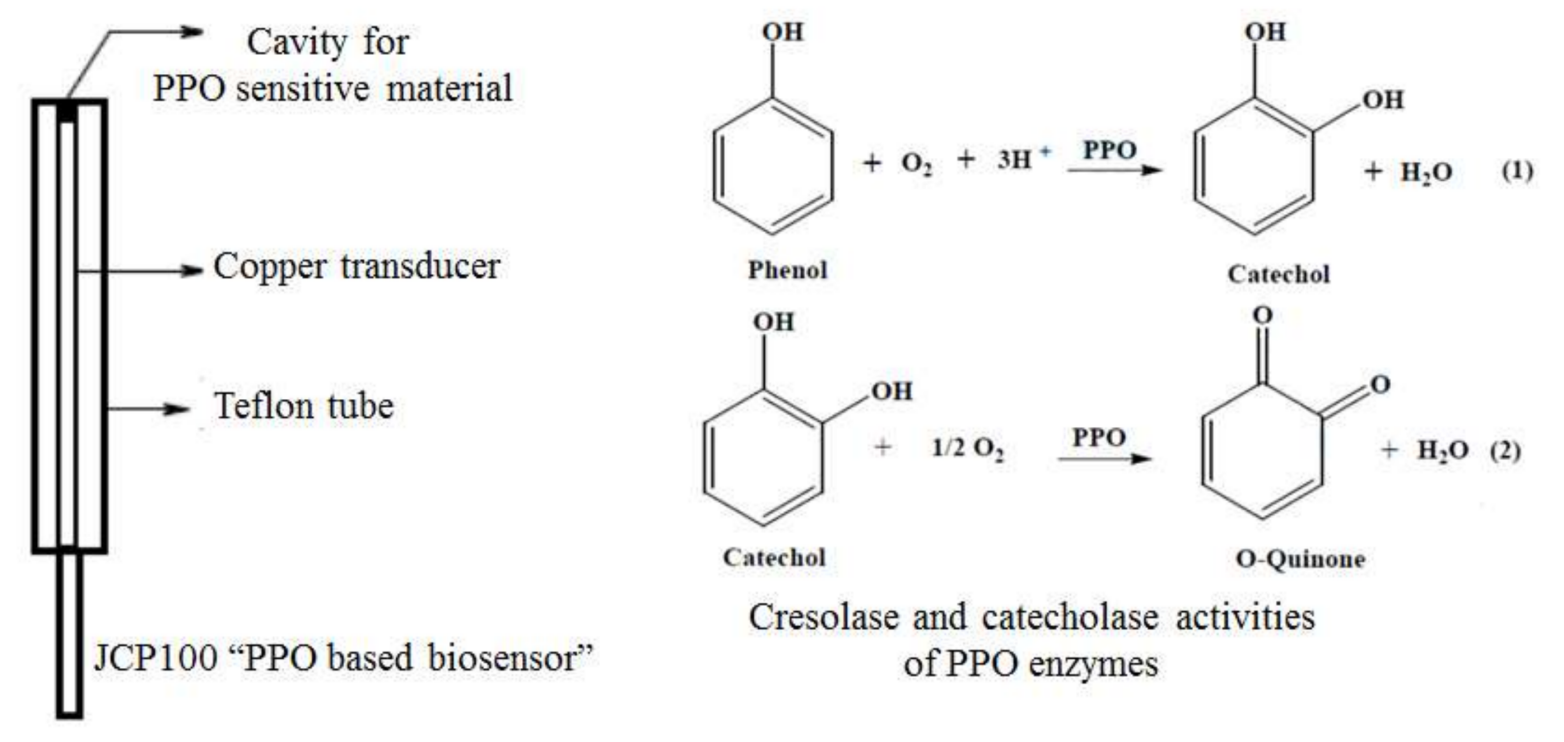
Biosensor configuration and related activities of polyphenol oxidase (PPO)-based biosensors.

The pastes were used to fill the cylindrical Teflon^®^ tubing serving as an electrode casing (Ø = 1 mm), that served as the electrochemical transduction device or working electrode (Scheme 1).

The effect of pH on the biosensor response against catechol was also evaluated for all systems, by using 0.1 M phosphate buffer solutions (PBS) in which the pH was adjusted from 3.0 to 9.0. The DPV assays were performed taking into account the following optimized parameters, namely: a pulse amplitude of 50 mV, a pulse width of 0.5 s, and a scan rate of 10 mV s^−1^. Prior to each DPV determination, the biosensor was submitted to 8 cyclic voltammetric scans from 0 to 1.0 V at 100 mV s^−1^ in order to get signal stabilization. The differential pulse (DP) voltammograms were background-subtracted and baseline-corrected, and then all data were analyzed and treated with the software Origin 8^®^ (OriginLab Corporation, Northampton, MA, USA).

### 2.5. Biosensor Applicability

The optimized biosensor was evaluated against 100 μM aqueous solutions of paracetamol, acetyl salicylic acid, methyldopa, and ascorbic acid standards in 0.1 M PBS, pH 7.0.

#### 2.5.1. Determination of Paracetamol in Tablets

In order to evaluate the suitability of the method for real samples, different categories of tablets were purchased from local drugstores (Table 2).

##### Sample Preparation

The samples were prepared accordingly to pharmacopeial procedures. Briefly, 10 tablets of each sample (Table 2) were crushed in a mortar, from which was taken a suitable amount of sample to prepare 1 mM stock solutions. The former solution was filtered and diluted till 100 μM, and this final solution was used in voltammetric (proposed method) and spectrophotometric (official method) assays. The measurements in UV–vis spectrophotometry (official method) were performed at 257 nm. All experiments were done in triplicates at room temperature.

### 2.6. Effect of Conditioning Time and Stability (Storage and Reuse)

The quickness of the biosensor response against 100 μM paracetamol solution was evaluated by varying the conditioning time prior to the electrochemical reduction in 10, 30, and 60–120 s.

The storage stability under 4 °C was checked weekly during 42 days for the same modified carbon paste, but conditioned in different devices, which were manipulated in different ways, thus avoiding temperature oscillation. Then, the biosensor response against 100 μM paracetamol solution was evaluated at the optimum conditions. 

The reuse of the same modified carbon paste conditioned in a single device was evaluated during 7 days at similar assay conditions, in order to check the impact of temperature oscillation.

The signal stability under repeated use was evaluated in six replications in the same day, by using freshly prepared biosensor.

### 2.7. Analytical Features: Linearity, Repeatability, Limit of Detection (LoD), and Recovery

The linear range and the linearity was determined (expressed by the regression coefficient (R^2^)), while the repeatability was expressed by the relative standard deviation (RSD). The accuracy and the recovery were assessed, taking into account the official method and the standard samples evaluation, which was obtained by means of linear regression equations [20,22,23].

### 2.8. Electrochemical Analysis and Statistical Analysis

Electrochemical analyses were performed using a μAutolabTipo III^®^ potentiostat/galvanostat integrated with GPES 4.9^®^ software (Eco-Chemie, Utrecht, The Netherlands). Measurements were performed using a 1.0 mL electrochemical cell (CP, JCP50, JCP100, or JCP200, respectively), platinum wire, and Ag/AgCl/KCl (3 M), representing the working electrode, auxiliary electrode, and reference electrode, respectively.

The statistical analyses of the data were performed using the BioEstat^®^ program, version 5.3. The statistical differences between groups were determined by the Tukey test, with *p* < 0.05 being considered statistically significant. For the construction of the graphics, the Origin 8^®^ program was used (OriginLab Corporation, Northampton, MA, USA). 

## 3. Results and Discussion

### 3.1. PPO Specific Activity and JEE Total Protein Activity

The PPO and total protein results of this work were obtained using a novel enzymatic extraction procedure using a Yononas^®^ frozen dessert maker to process the fruit in frozen form, thereby preserving the enzymatic activity.

The PPO activity value obtained was 616 U/mg protein and the total activity value was 1795 U/mg protein/100 μL, which was slightly higher than those calculated for eggplant (*Solanum melongena*) pulp (376 U/mg protein) and its seed (500 U/mg protein) [26,27,28].

### 3.2. Biosensor and Assay Optimization

**Figure 1:**
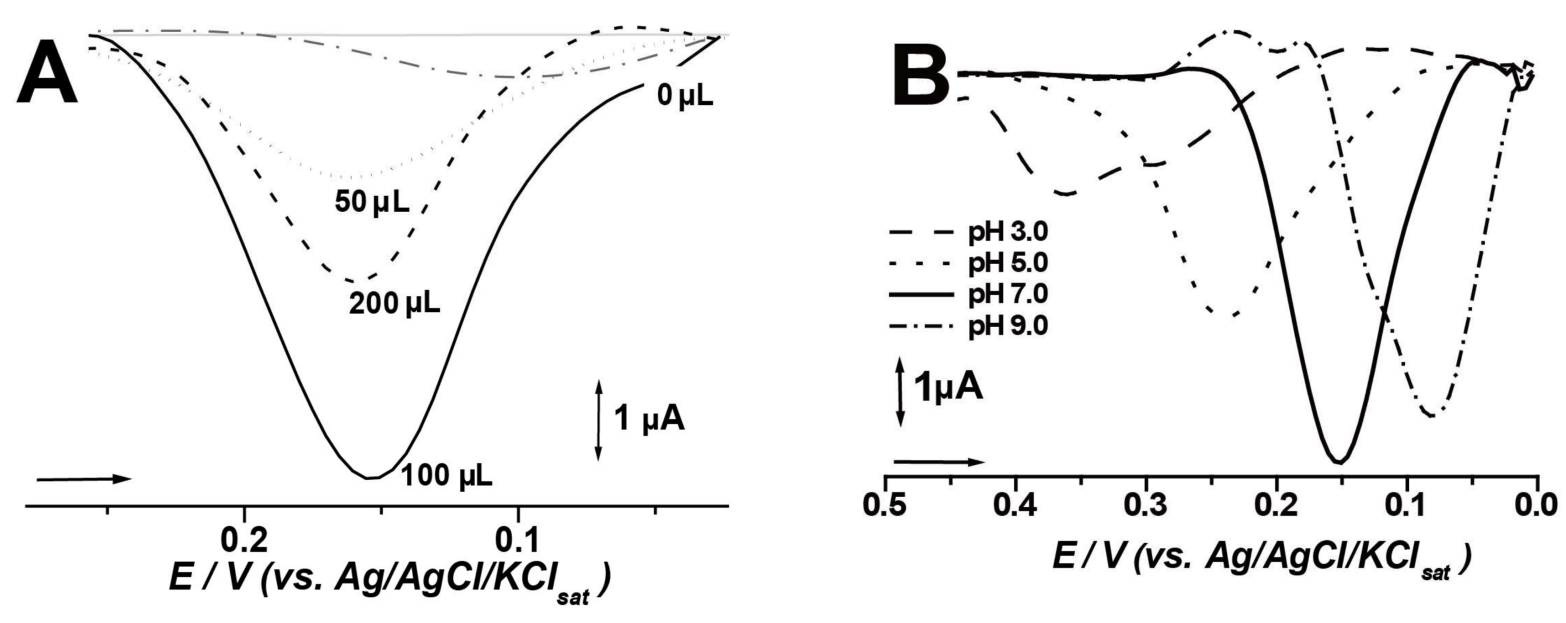
(**A**) DP voltammograms obtained for 0.1 mM catechol in pH 7.0, 0.1 M phosphate buffer (PB) solution for biosensors produced with different additions of JEE per 100 mg of carbon paste (the grey line is the blank). (**B**) JCP100 biosensor response for 0.1 mM catechol solution in different 0.1 M PB solutions and pH values.

In order to ascertain the optimal proportion of enzymatic crude extract perunit carbon paste, the biosensor performance was evaluated against catechol, which is the best probe for polyphenol oxidases. It was found that the highest response was achieved when 100 μL of JEE was added to produce ca. 100 mg of carbon paste-based biosensor (Figure 1A). Therefore, it can be inferred that smaller proportions will not offer highest enzymatic activity, whereas higher amounts may not produce any gain, exerting a negative effect on the electrochemical properties, probable due to the non conducting nature of bioorganic materials. For instance, it was found that 616 U/mg of protein/100 μL of JEE delivered the best results [12,13,29]. Owing to the great relevance of pH in enzymatic activity and redox processes of organic compounds, the biosensor, herein named JCP100, was evaluated in different pH conditions. The highest activity was observed at pH 7.0 (Figure 1B). This value is in agreement with some literature reports for PPO-based biosensors [4,30].

Therefore, all subsequent assays were performed with the JCP100 biosensor at neutral pH 7.0.

### 3.3. Biosensor Activity against Phenolic Drugs 

**Figure 2:**
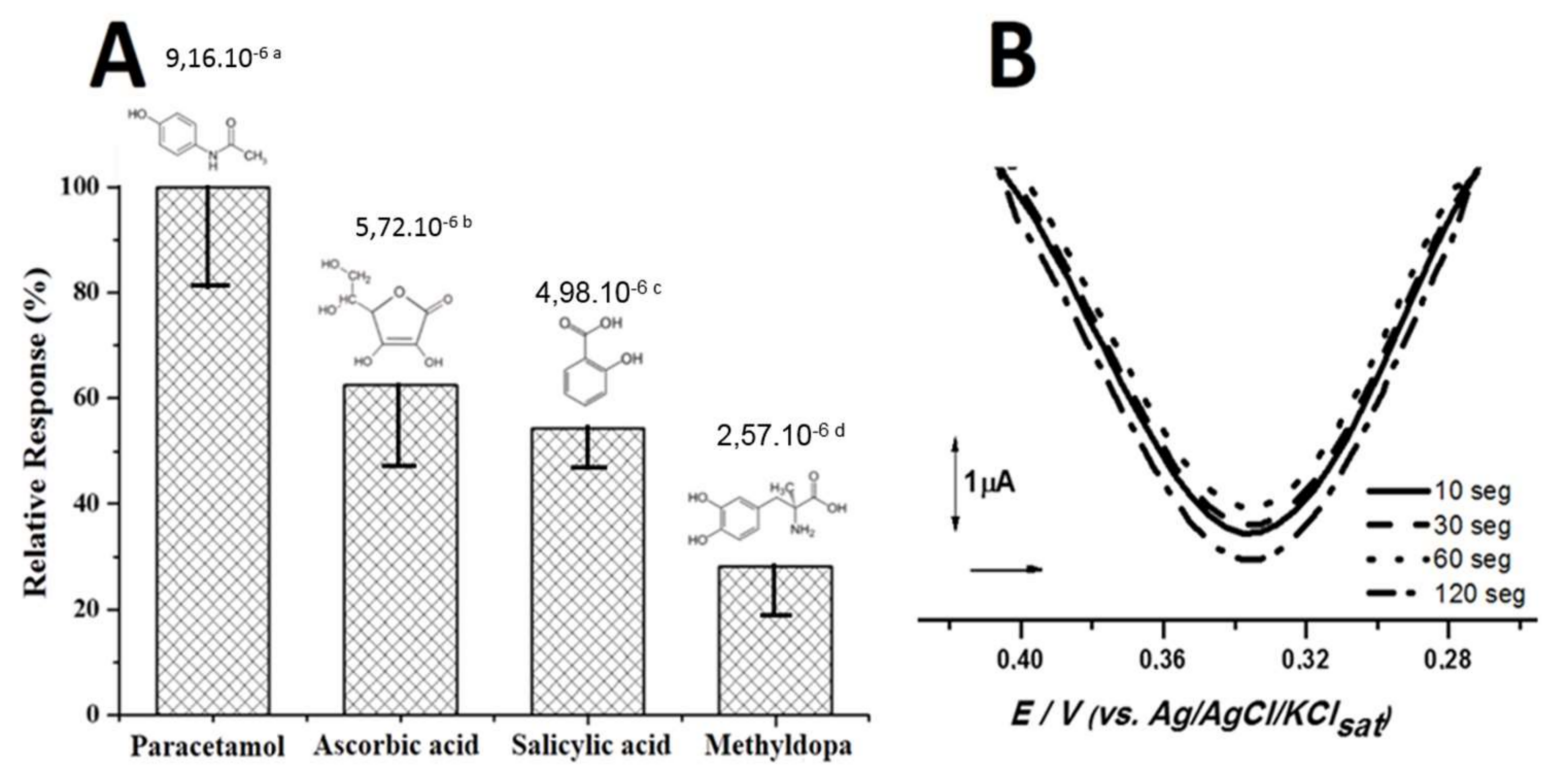
(**A**) The relative response of the JCP100 biosensor observed against different 100 μM concentrations of the various drugs evaluated. The maximum current response was stated as the relative response. (**B**) Time response of JCP100 biosensor observed for 100 μM paracetamol in pH 7.0, 0.1 M PB solution. Data followed by the same superscript are not significantly different (*p* > 0.05) by ANOVA and Tukey test.

The performance of the JCP100 biosensor was evaluated against ascorbic acid and the phenolic drugs paracetamol, salicylic acid, and methyldopa (Figure 2A). We did not observe any statistical differences between the groups using ANOVA and the Tukey test (*p* > 0.05). The cathodic peak currents obtained for 100 µM paracetamol was approximately −25 µA, which was 10 µA higher compared with the other drugs at equal concentration. Thus, the biosensor’s time response for this drug was evaluated by varying the conditioning time prior to DPV scanning. The time required to achieve steady-state current in the presence of paracetamol was 2 min (Figure 2B). 

**Figure 3:**
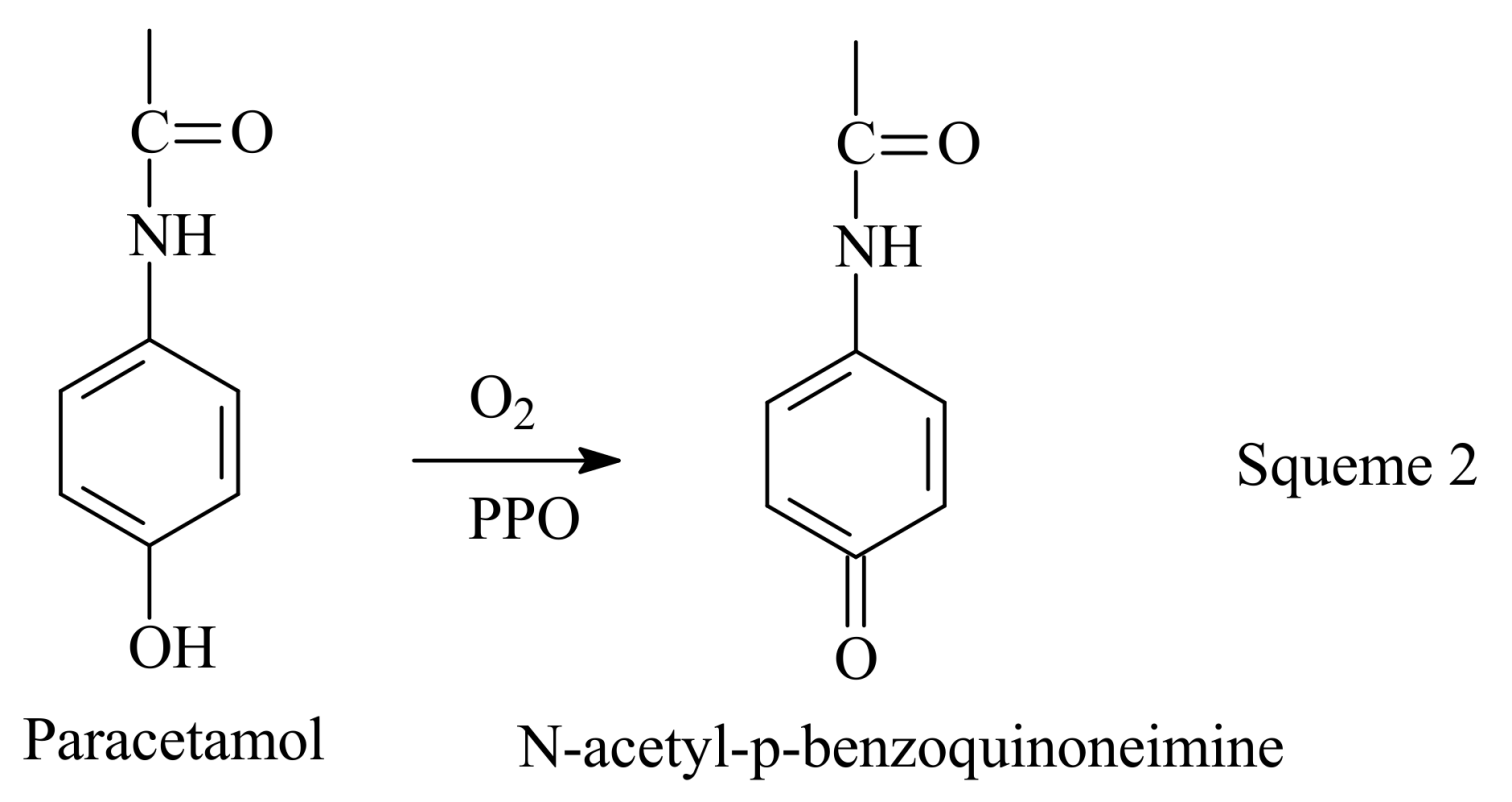
Biochemical mechanism of PPO on PCT oxidation.

Nevertheless, the increment observed for cathodic currents from 0 to 2 minutes was less than 1 µA. Thus, it can be inferred that the immobilized system exerts the PPO activities (Scheme 1) with a fast response time. Indeed, the amperometric PPO-based biosensors exert their action by converting phenolic compounds into quinone derivatives (Figure 3, Scheme 1), which can be electrochemically reduced at lower peak potentials. This mechanism avoids higher overpotentials, which are commonly required in anodic processes for monophenolic species, thus reducing expressively the number of interfering compounds [3,5].

**Figure 4:**
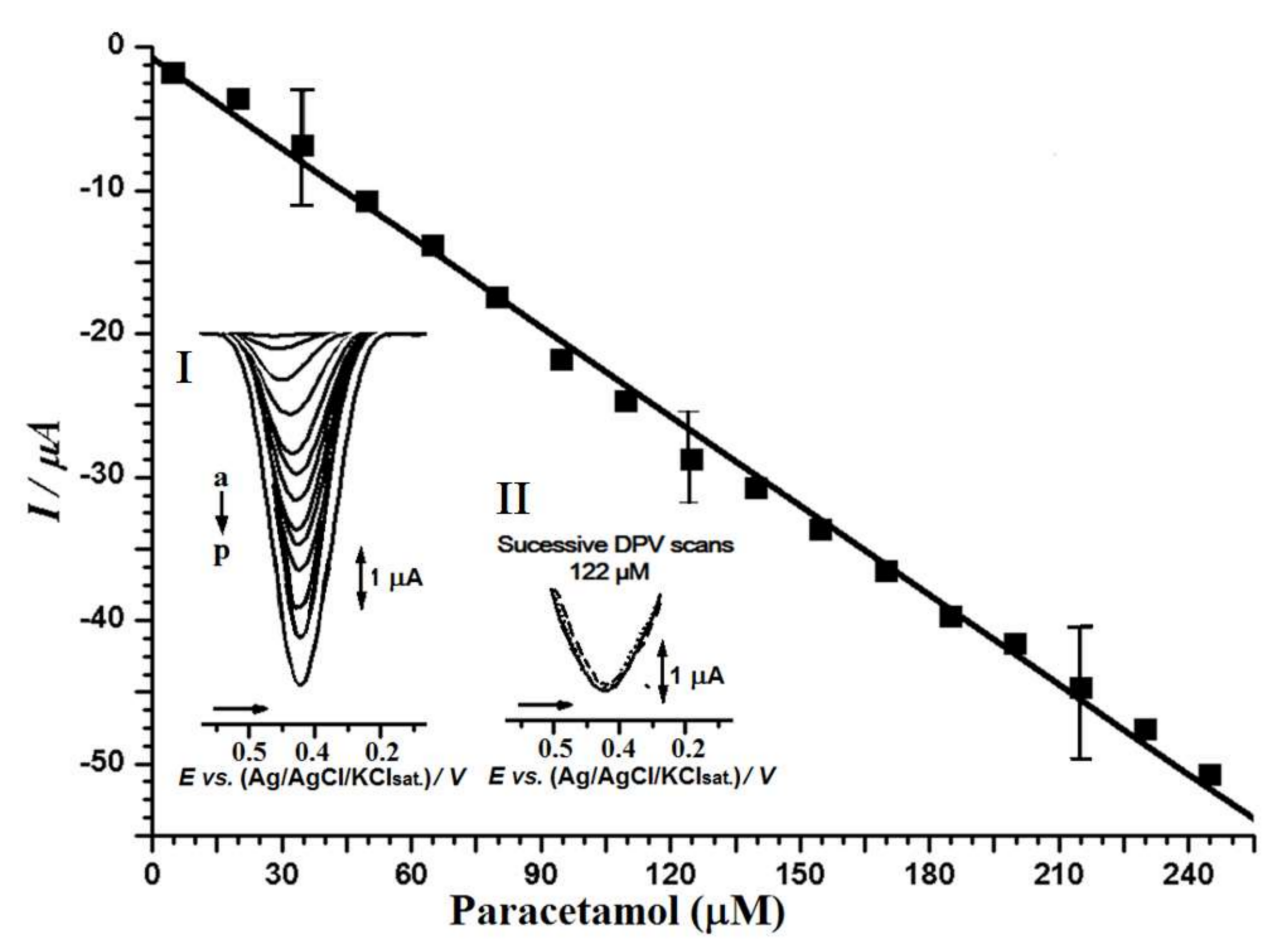
Calibration graph obtained for increasing (a→p) concentrations of paracetamol in 0.1 M PBS, pH 7.0 with the JCP100 biosensor. Inset: (I) The related DP voltammograms and (II) the successive scans for 122 mM. Other parameters are outlined in Section 2.5.

A calibration curve was constructed and calculated for the analysis of paracetamol using the JCP100 biosensor. A linear relationship between the peak currents and paracetamol concentrations was obtained from 5 to 245 μM (R = 0.9994), with a detection limit of 3 μM (Figure 4). The relative standard deviation (% RSD) at different levels (low, medium, and high) was below 5%, being of 3.2% for triplicate determinations in a 122 μM paracetamol solution (Figure 4 inset II), *p* = 0.0013 (Tukey test, 95%).

In order to establish comparisons with other vegetable PPO-based biosensors proposed for paracetamol analysis, the analytical data in this study were compared with other studies from literature as provided in Table 3.

As can be seen, the solanaceae jurubeba is a promising source of PPO enzymes, being that the biosensor response was higher than the one observed for other devices. Hence, in order to evaluate the suitability of reuse and storage conditions, the stability of the paste prepared to construct the JCP100 biosensor was stored in individual packages and a single package. In both cases, the pastes were stored at 4 °C temperature and retrieved minutes before use to reach room temperature (20 ± 2 °C), after which the analyses were performed.

**Figure 5:**
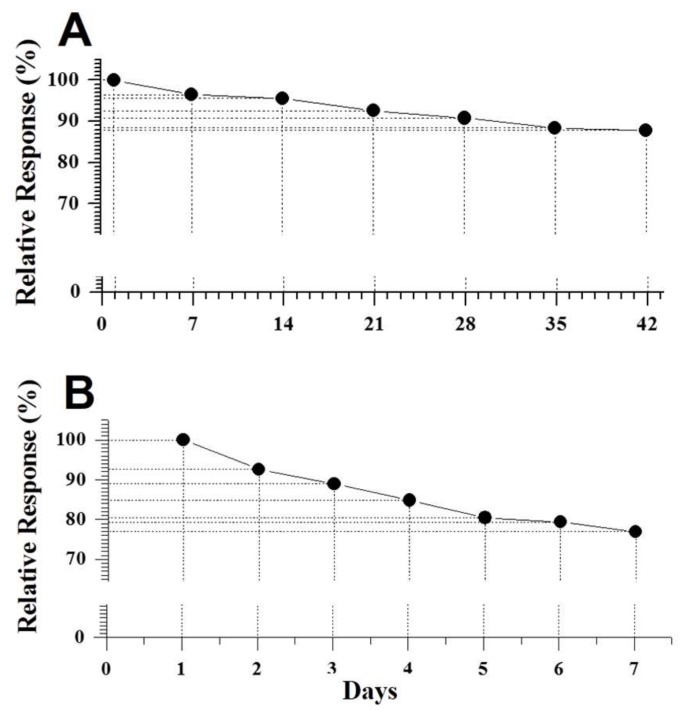
Relative response resultsobtained forthebiosensor stability under single-use packages (**A**) and multiple-use packages (**B**).

The first attempt was performed in order to avoid temperature variations; whereas the second for practical reasons and sensor robustness. The individual packages were monitored a single time during six weeks. At the end of day 42, the resulting JCP100 biosensor delivered 87.79% of its initial response. The cathodic peak currents obtained for 100 μM paracetamol fell from 2.29 to 2.00 μA (Figure 5A). The Tukey test (95%) was also used in the parameter evaluation, and demonstrated that the results obtained in the detection of paracetamol at different times are statistically the same, with *p* = 0.0251.

In turn, the JCP100 stability was also investigated for pastes stored in a single package over a period of seven days. Owing to the repeated manipulation, leading to temperature variations, at the end of the seventh day, the signal was only 77% of the one observed at time zero (Figure 5B).

### 3.4. Pharmaceutical Analysis and Recovery Assays 

In order to evaluate the suitability of the JCP100 biosensor for pharmaceutical analysis, the biosensor performance was evaluated in real samples of different categories (Table 2), and the results compared with those of the official method (Table 4).

The paired *t*-test (Tukey 95%) was then applied to verify if there was a difference between the results obtained in the detection of paracetamol in the actual samples when comparing the official and proposed methods. The results showed that there is no statistical difference between the values obtained.

Moreover, all samples were in accordance with the recommended pharmacopeial values, in which the drug potency must be between 95.0% and 105.0% [21]. 

The accuracy and precision of the proposed method were also evaluated, and the recovery assay for different concentrations is presented in Table 5.

Recoveries calculated for the results shown in Table 5 revealed a relative error ranging from 0 to 2.57%, which is less than the 5% limit value. Therefore, the proposed DPV method indicates that it is in strong agreement with the pharmacopeial method. Thus, it can be successfully applied for the determination of paracetamol in such a dosage form without any pretreatment processing, offering low costs and faster development.

## 4. Conclusions

The crude enzymatic extract obtained from the *S. paniculatum* L. fruit was shown to be an efficient source of PPO, which was successfully applied inthe development of carbon paste-based biosensors for paracetamol determination in medicines. 

The proposed biosensor exhibited good repeatability and satisfactory stability as well as suitable sensitivity and selectivity. It was established that the CP/JCP100 biosensor has a linear range of 5 to 250 μM for paracetamol analysis and a detection limit of 3 μM. Moreover, the low cost, simplicity, and fast production highlighted the attractiveness of this alternative device in analyses and quality control of pharmaceutical formulations.

## Figures and Tables

**Table 1:** Biosensor compositions consisting of different proportions of JEE in carbon paste (CP).

Biosensor	Graphite Powder (mg)	Vegetable Extract (μL)	Mineral Oil (mg)
CP	100	-	30
JCP50	100	50	30
JCP100	100	100	30
JCP200	100	200	30

**Table 2:** Identification of the commercial samples analyzed by the jurubeba biosensor.

Samples	Tablets
1	Reference 750 mg
2	Generic 750 mg
3	Similar 750 mg
4	Reference 500 mg
5	Generic 500 mg
6	Similar 500 mg

**Table 3:** PPO carbon paste-based biosensors for paracetamol pharmaceutical analysis. LoD: limit of detection.

PPO Plant Source	Enzymatic Activity of PPO (U/mg Protein)	Linear Range (μM)	LoD (μM)	References
*Persea americana*	375	1200–53,000	880	[3]
*Cucurbita pepo*	137	1200–53,000	690	[30]
*Solanum melongena*	552.60	20–200	5	[5]
*Solanum paniculatum* L.	616	5–245	3	This work

**Table 4:** Evaluation of the JCP100 biosensor performance for different commercial tablet samples of paracetamol (*n* = 3).

Medicines Category	Labeled Value (mg)	Official Method (mg)	Proposed Method (mg)	Relative Error * (%)	Relative Error ** (%)	Relative Error *** (%)
Reference _1_	750	773.76 ± 1.20	769.74 ± 0.77	+3.16	+2.63	−0.51
Generic _2_	750	752.94 ± 0.77	747.06 ± 0.36	+2.94	−0.39	−0.78
Similar _3_	750	753.86 ± 0.59	751.84 ± 0.65	+0.51	+0.24	−0.26
Reference _4_	500	510.38 ± 0.46	507.18 ± 0.40	+2.07	+1.43	−0.62
Generic _5_	500	497.36 ± 0.34	495.38 ± 0.37	−0.52	−0.92	−0.39
Similar _6_	500	505.60 ± 0.38	491.46 ± 0.58	+1.12	−1.70	−2.79

_1_ *p* = 0.0287; _2_ *p* = 0.0069; _3_ *p* = 0.0062; _4_ *p* = 0.0126; _5_ *p* = 0.0030; _6_ *p* = 0.0011 (Tukey 95%). * Relative error: relative to the tabulated value and the result obtained by the official method. ** Relative error: referring to the tabulated value and the result obtained by the proposed method. *** Relative error: referring to the results obtained between the proposed method and the official method.

**Table 5:** Recovery assay for standard addition of different concentration levels (*n* = 3).

Paracetamol Concentration (μM)				
Tablet	Incorporated	Found	RSD (%)	Recovery (%)
Reference	0	0	0	100
	10	9.76 ± 0.13	2.40	97.60
	20	19.71 ± 0.67	1.45	98.55
	30	29.56 ± 0.88	1.46	98.53
	40	40.06 ± 0.91	0.15	100.15
Generic	0	0	0	100
	10	10.12 ± 0.23	1.20	101.20
	20	20.03 ± 0.97	0.15	100.15
	30	29.97 ± 0.33	0.10	99.90
	40	41.03 ± 42	2.57	102.57
Similar	0	0	0	100
	10	9.96 ± 1.14	0.40	99.60
	20	20.14 ± 0.31	0.70	100.70
	30	30.02 ± 0.98	0.06	100.06
	40	39.91 ± 0.77	0.22	99.77
